# Social information affects adults’ evaluation of fairness in distributions: An ERP approach

**DOI:** 10.1371/journal.pone.0172974

**Published:** 2017-02-24

**Authors:** Mitsuhiko Ishikawa, Yun-hee Park, Michiteru Kitazaki, Shoji Itakura

**Affiliations:** 1 Department of Psychology, Graduate School of Letters, Kyoto University, Yoshidahonmachi, Sakyo-ku, Kyoto, Japan; 2 Department of Computer Science and Engineering, Toyohashi University of Technology, Tempaku-cho, Toyohashi, Japan; IRCCS Istituto Auxologico Italiano, ITALY

## Abstract

The sense of fairness has been observed in early infancy. Because many studies of fairness in adults have used economic games such as the Ultimatum Game, it has been difficult to compare fairness between adults and infants. Further, recent studies have suggested that social information about actors who behave fairly or unfairly may influence the judgement of fairness in infants. Therefore, to compare the sense of fairness between infants and adults, the study using paradigm in infant research is required. We examined how social information about two characters, either prosocial or antisocial, affects the event-related potential response (ERP) to fair or unfair resource distributions in adults. In the habituation phase, participants were informed about characters’ social information through their actions. One character then distributed resources fairly or unfairly, and ERP was measured at the end of the distribution. Data from eighteen adult participants were analysed. A significant interaction of social information and fairness was found for late positive potential (LPP), but a post-hoc t test revealed a significant difference between fair and unfair conditions only for actions of the antisocial character. We found that LPP can reflect the sense of fairness affected by social information. Comparison with infant studies suggests that the sense of fairness may change during development.

## Introduction

The concept of fairness is one of the crucial components of morality and is an essential factor in the maintenance of social groups. People prefer equitable distributions of resources over unequal distributions and are highly sensitive to their own disadvantages [1]. This has been examined using a social psychological approach, for example using the Ultimatum Game (UG) [2]. Developmental studies have revealed that human infants possess a sense of morality in early life.

Hamlin, Wynn & Bloom [3] examined social evaluation in preverbal infants. Two characters, a “helper,” who helped another character to achieve a goal, and a “hinderer,” who prevented another character who was attempting to achieve a goal, were presented in the habituation phase that was intended to provide social information to young infants. Measurement of preferential looking times revealed that three month olds preferred a prosocial character over an antisocial character. These results suggest that infants are already able to form social preferences (see also [4]).

Using the same animations, Cowell & Decety [5] used electroencephalography (EEG) to examine the neural responses of children between the ages of 12 and 24 months as they observed a character who either helped or hindered. Amplitudes of average voltages for the parietal electrodes (Pz) in the 300–500 ms range were greater for prosocial scenes than for the antisocial scenes. This event-related potential (ERP) component, P400, has been said to be an infant version of the adult N170-N200 [6] [7], a response associated with the social categorization process in adults [8]. Another developmental study also found that amplitudes of the P400 component after habituation were greater when the prosocial agent appeared [9]. These results suggest that P400 differences predict children’s preference for prosocial over antisocial characters. Taken together, this evidence suggests that the processing of social information can affect later judgement of characters, and the judgement of characters can be reflected in infant P400, which is reflected similar in function to the adult N170-N200 ERP component.

Prosociality has been examined with animations depicting fair and unfair distribution situations. Meristo & Surian [10] used animations of distributions of strawberries to familiarize participants with two characters as fair or unfair donors. They argued that ten-month-old infants can perceive unfair distributions as negative as the antisocial actions such as hitting others in. Thus an unfair distribution may be an example of an immoral action that requires only a simple judgement of fairness.

The Ultimatum Game (UG) paradigm has been used to examine blood oxygen responses to unfair offers [11]. The original UG is conducted by two players, a responder and proposer, and the proposer is asked to divide money between them [12]. While the proposer can adjust the amount of money that is given to the responder, the responder is only able to accept the proposal or reject it and receive nothing. In this context, rejections of unfair offers may be thought as responses to norm violations [11]. In a functional Magnetic Resonance Imaging (fMRI) study, the insula and the dorsal medial prefrontal cortex were activated in the condition receiving unfair offers [11]. Thus these brain regions may be associated with fairness and may reflect violations of social norms. However, as the authors pointed out, this result may have been affected by the decision-making processes in the evaluation of unfairness. Qu, Wang & Huang [13] conducted an ERP study of the UG and examined judgements of fairness. Fair offers elicited a larger P300, a component evoked around 300ms after stimulus presentation, relative to unfair offers. P300 has been said to be related to affective evaluation and positive or negative stimuli judgements [14] [15]. Another ERP component, the late positive potential (LPP), which is observed approximately 300 to 400ms after stimulus onset and persists for nearly 1000 ms [16], may reflect a function similar to P300 [17] [18]. Although both P300 and LPP components can be observed with the same tasks, LPP has been implicated in the higher cognitive process of social evaluation based on contextual information [19]. Specifically, in moral judgement studies, LPP not only reflected judgement of actions as prosocial or antisocial but also was sensitive to personality differences and social context [20] [21].

Recent studies of fairness have begun to focus on the effects of social information and context on moral judgement [22] [23]. However, in many such studies, it has been difficult to examine only the perception of fairness using social information, because the UG includes participants’ own monetary rewards. [11] [13]. Given the results of developmental studies, it is evident that social information about characters can be provided by means of animations, [3] and participants can judge fairness by watching actors behave either fairly or unfairly in distributions [10]. Using animations to investigate fairness can also help to compare the sense of fairness in adults and infants.

The present study used animations similar to those employed in infant studies to investigate how social information affects the perception of fairness and unfairness in adults, as reflected in ERPs. Social information about a prosocial Helper and an antisocial Hinderer was given before the distribution animations; thus participants would have expectations about how these characters should act. When fair or unfair distributions were conducted, early components, such as P300, should be affected by social evaluation based on these expectations. Because N170-N200 components in adults may reflect responses similar to the P400 in infants, we examined components in this time window. We also examined LPP, which, as a component reflecting higher cognitive functions, may index the judgement of fairness based on social information.

## Materials and method

We assure our experimental protcol was approved by the Research Ethics Review Board at the Department of Psychology, Kyoto University, Kyoto, Japan, and all participants gave their written informed consent to participate in this study.

### Participants

Twenty-seven students (16 males, 11 females; mean age 22.19±3.8 years) were recruited. Nine participants were excluded from the analysis because of high artefact rates, including many body movements and eye blinks. As a result, 18 participants (12 males, 6 females) provided reliable data for analysis.

### Procedure

**Fig 1:**
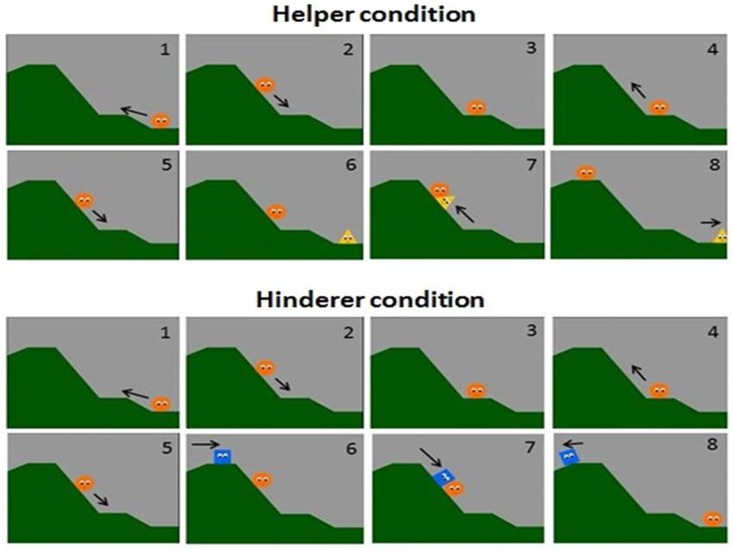
Animations in the habituation phase (see also Hamlin et al., 2007).

The animations were presented in two phases, a habituation phase and a test phase. At the beginning of the study, participants were told to watch the animations during habituation phase. In the habituation phase (Fig 1), participants were told simply to watch the animations. Ten animations describing a character who helped or hindered another character to achieve a goal were presented. In the first habituation animation, a character (called the ‘climber’) at the bottom of hill was shown. The climber made repeated attempts to climb the hill, suggesting to participants that the climber needed to be helped to reach the top of the hill. During the habituation task, either a Helper who pushed the climber from behind or a Hinderer who pushed down on the climber from above appeared. Because of their actions, the climber was shown either reaching the top of the hill or giving up the climb. All animations in the habituation phase were 22 seconds long.

**Fig 2:**
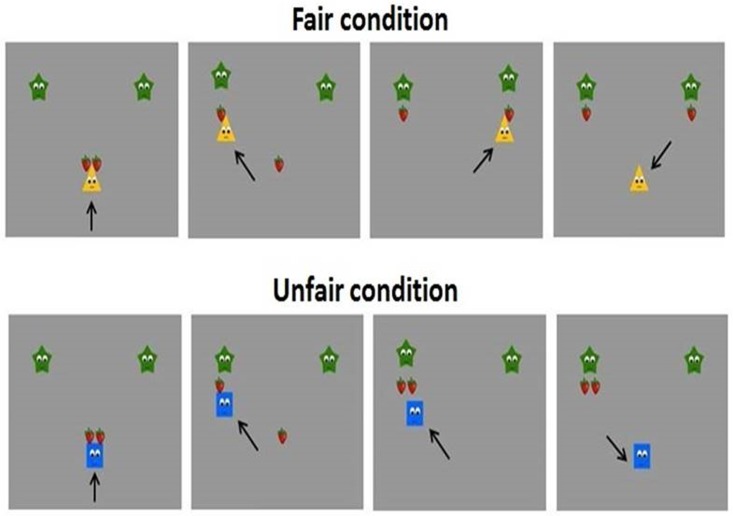
Animations in the distribution phase (see also Meristo & Surian, 2014).

After the habituation phase, participants were instructed to count the number of fair trials and not to move as much as possible. In the test phase (Fig 2), three characters, including a Helper or a Hinderer, were presented. The test animations consisted of two distributions conducted by either a Helper or Hinderer. First, two stars, the receivers of the distributions, were presented, one on the upper left side of the screen and the other on the upper right side. Then the Helper or Hinderer appeared with two strawberries, and remained in the lower centre of screen for four seconds. After that, an agent, the Helper or Hinderer, began the distribution of the two strawberries to the stars. The Distribution phase included two fairness conditions (Fair, Unfair), with each agent performing either a fair distribution, giving one strawberry to each star, or an unfair distribution, giving all strawberries to the same star. To ensure that participants concentrated on the animations, they were required to count the number of fair distributions over all the trials. We analysed ERP data from the onset at the end of the second distribution. Each animation was 18 seconds long.

Each participant completed total 160 trials, 40 trials per condition (Helper Fair; Hinderer Fair; Helper Unfair; Hinderer Unfair), presented in random order. Each block was composed of 40 trials (10 trials per condition), and participants took a rest during the period between blocks if they felt tired. After completing all trials, participants were told the purpose of the study. The entire session lasted about 150 minutes.

### Apparatus

Visual stimuli were presented on a 20-inch A1081 Apple Cinema Display (Apple, USA). The order of events was controlled by a program written using E-prime 2.0 (Psychology Software Tools).

### EEG data

EEG signals were recorded by a biological amplifier (TEAC Polymate II AP216, 16channel, 16 bit, up to 1000Hz sampling) using 9 scalp electrodes (F3, Fz, F4, C3, Cz, C4, P3, Pz, and P4) according to the extended 10–20 system, with two earlobe electrodes. The reference was average of the left and right earlobes. The electrode impedance was kept below 10 kΩ. The EEG was recorded at a sampling rate of 1000 Hz and band pass filtered between 0.5 and30 Hz. The ERPs in each condition were averaged separately with an epoch beginning at the end of the distributions event and continuing for 1000 ms. The baseline was corrected using the pre-stimulus mean voltage. Trials containing eye blinks, eye movements, or muscle potentials exceeding ±50μV were excluded from the average for each electrode. Participants who produced artefacts on over 10% of all trials were excluded from analysis. The mean amplitude data for N200 was segmented from 120 to 200 ms after the end of the distribution. P300 was investigated using the average amplitude during a time range from 250 to 500 ms, and LPP was segmented ranging from 820 to 890 ms from onset.

## Results

**Fig 3:**
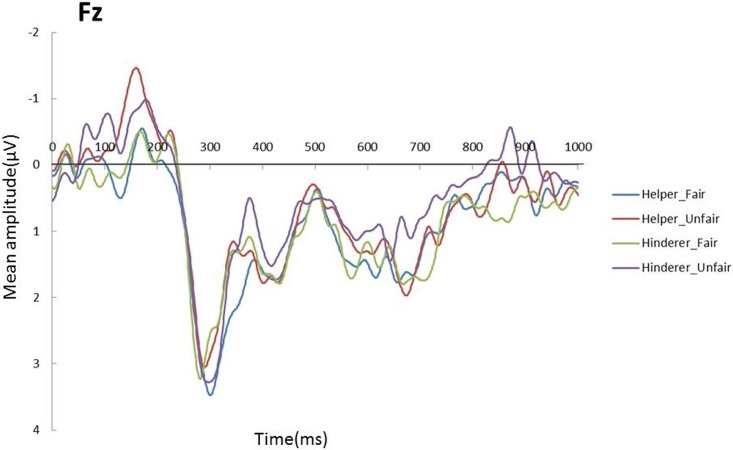
Grand mean ERPs on Fz elicited by the end of distributions for each of the four conditions.

**Fig 4:**
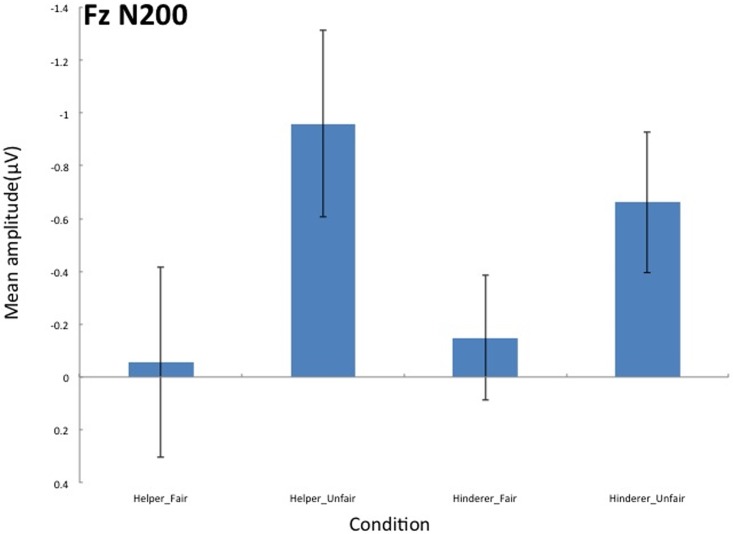
Grand mean N200 components on Fz elicited by the end of distributions for each of the four conditions.

Multiple comparisons were performed with the Holm correction. A 2 × 2 ANOVA with two levels of Social information (Helper, Hinderer) and two levels of Fairness (Fair, Unfair) was conducted on the mean N200 amplitude for each electrode. A significant main effect of Fairness was observed for Fz (*F* (1,17) = 9.0431, *p* = .0296, *η*_*p*_^*2*^ = .2492) (see Figs 3 and 4), with the amplitude of the Unfair condition more negative than that of the Fair condition.

**Fig 5:**
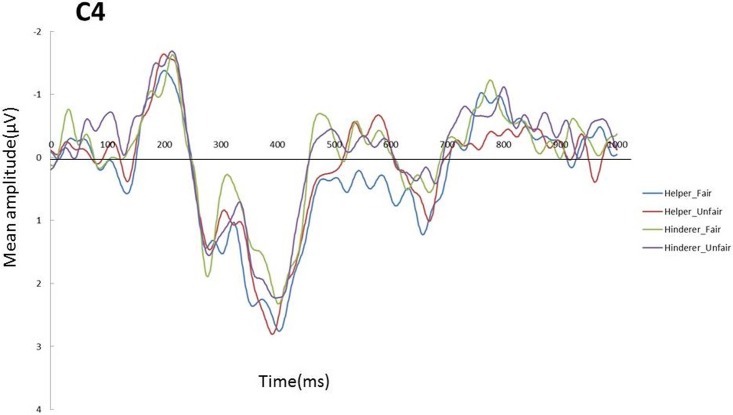
Grand mean ERPs on C4 elicited by the end of distributions for each of the four conditions.

**Fig 6:**
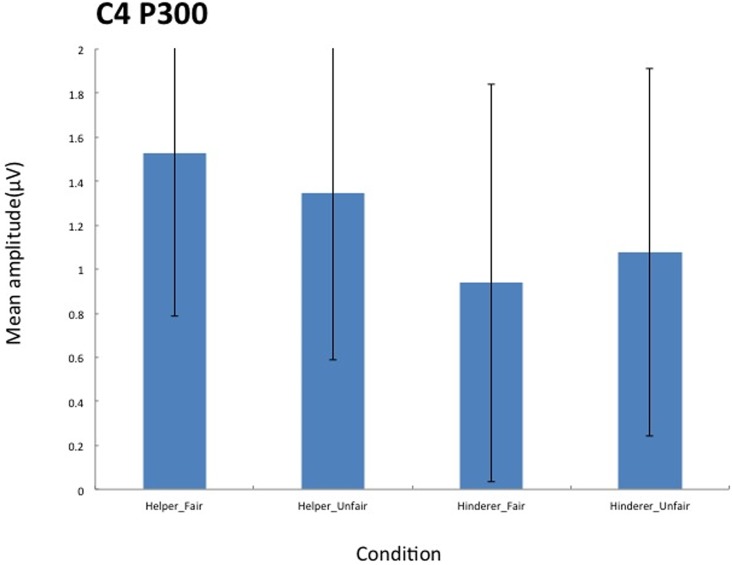
Grand mean P300 components on C4 elicited by the end of distributions for each of the four conditions.

A 2 × 2 ANOVA with two levels of Social information (Helper, Hinderer) and two levels of Fairness (Fair, Unfair) was also conducted on mean P300 amplitude for each electrode. The main effect of Social information was significant for C4 (*F* (1,17) = 5.06, *p* = .038, *η*_*p*_^*2*^ = .23), with greater amplitude for the Helper condition than for the Hinderer condition (see Figs 5 and 6).

**Fig 7:**
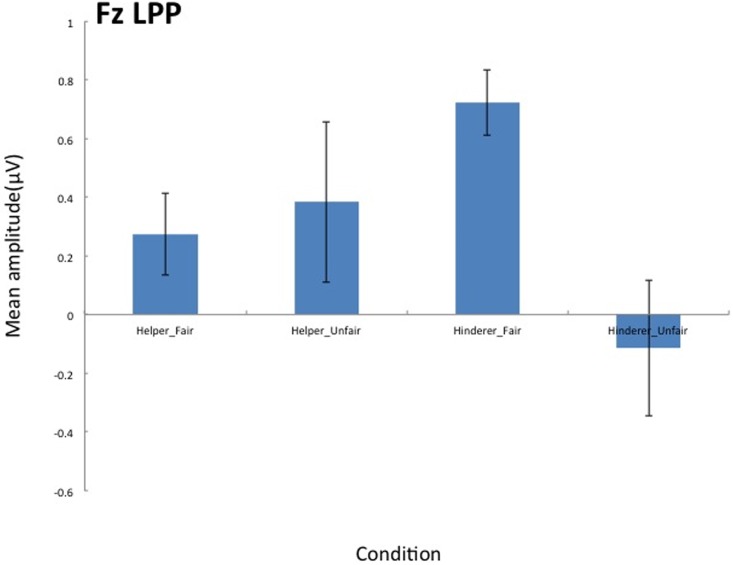
Grand mean LPPs on Fz elicited by the end of distributions for each of the four conditions.

A similar 2 × 2 ANOVA was conducted on mean amplitude of LPP for each electrode. The interaction of Social information and Fairness was significant for Fz (*F* (1,17) = 3.98, *p* = .0623, *η*_*p*_^*2*^ = .19) (see Fig 3). Post-hoc t-tests corrected by the Bonferroni method indicated greater amplitude for the Hinderer-Fair condition than for the Hinderer-Unfair condition (*F* (1,17) = 8.92, *p* = .0083, *η*_*p*_^*2*^ = .34) (see Fig 7).

## Discussion

We expected that judgements of the character’s role would be reflected in N200 and P300, and that LPP would demonstrate the higher cognitive process of fairness, which is dependent on both social information and distributions. There are three principal findings. First, N200 was significantly affected by fairness. Second, a significant main effect of social information was found for P300, and third, a significant interaction of social information and fairness for LPP. A comparison of these results with infant studies suggests that the sense of fairness may show developmental changes.

Based on studies suggesting that infant P400 and adult N200 may have similar functions in response to identical stimuli [24] [25] [26], we analysed the N200 ERP component. Although infant P400 appears to occur with the evaluation of a character’s social information [9], the present results showed only a difference between fairness conditions. N200 has been used to index cognitive control, novelty, and expectancy violation [27] and has been shown to reflect the motivational significance of negative outcomes [28]. In a study using UG [29], N200 amplitudes were different between the recognition of fair and unfair offers. Because the UG depends on judgements of fairness in deciding whether to accept or refuse proposals, participants should concentrate on assessing whether the offers are fair or unfair. In the present study, we instructed participants to count the number of fair trials. This procedure could have motivated participants to judge the fairness of distributions, and, as a result, N200 amplitude reflected the fairness conditions. Although we too found that the N200 component can be an index of fairness, social information did not have significant effects. Thus N200 may index only whether the immediate situations are fair or not, regardless of contextual information.

N200 and infant P400 showed different functions, while our P300 result reflected the same function as P400 in infant studies [4] [5]. Classical ERP studies have also suggested a similarity between P300 and P400 [30] [31]. Thus P300, like P400, may distinguish whether the distributing character is a Helper or Hinderer by means of social information about the character. However, the P300 response is also considered an index of contextual and semantic expectation [32]. In our paradigm, participants could generate expectations from social information in the habituation phase, and they may have expected that the Helper would distribute strawberries fairly. Therefore, P300 should have been evoked when the Helper distributed unfairly or the Hinderer distributed fairly. A problem with the paradigm may explain our results. We did not place the habituation phase in between the test trials, but presented social information only before the test phase. Thus participants may have acquired social information from the adjacent results of the characters’ distributions. P300 components may therefore have been disrupted within test trials. However, our findings suggest that the P300 response in adults reflects the judgement of social character. Further studies in which this paradigm is modified by inserting habituation phases in the interval between distributions are necessary to explain the effect of social information on the P300 component.

We predicted that LPP would reflect the highly cognitive process of using social information and a sense of fairness. As we predicted, only LPP showed a significant interaction, which revealed that the distributions violated the expectation that the Helper would behave prosociality and the Hinderer would act antisocially. LPP is usually observed in response to evaluative incongruence [32] and trait inconsistencies [33]. Our results suggested that LPP can be observed in a moral judgement situation involving social information about a character acting either fairly or unfairly. However, the results of the post-hoc t test indicated only a significant difference between the Hinderer-Fair condition and the Hinderer-Unfair condition. This result may reveal the importance of characters in studies about fairness or morality and differences in the development of a sense of fairness.

Surian, Meristo, Ueno, & Itakura (submitted) examined fairness in 15-month-old infants using the same animations as the present study, both in the habituation phase and the distributions phase. This study measured looking time in the distributions. Results showed that infants looked longer at the Helper-Unfair condition than at the Helper-Fair condition, and there was no difference between Hinderer conditions. This result suggests that infants expected the prosocial character to make fair distributions, but they may not have generated expectations about the Hinderer, the character showing antisocial actions, because it was evaluated as undesirable. Kanakogi, Okumura, Kitazaki & Itakura [34] reported that preverbal 10-month-old infants show rudimentary sympathetic behaviour with interpretation of social evaluation. In Hamlin, Wynn & Bloom [3], 6-month-olds were attracted to prosocial individuals and avoided antisocial individuals; 3-month-old infants showed the same tendency [35]. These previous studies suggest that infants are strongly influenced by social information, especially about prosociality. Therefore, in Surian, Meristo, Ueno, & Itakura, (submitted), infants’ expectations were violated when the prosocial character acted unfairly. On the other hand, the present results indicated that adults may not have strongly expected that the preferable prosocial character would conduct distributions in accordance with fairness. This difference between adults and infants suggests that the sense of fairness that depends on social information can be influenced by developmental factors. Kanakogi [36] pointed out that adults have more experience, such as social interactions and media exposure, that could influence the evaluation of social information. Therefore, adults and infants may have different biases about the antisocial character. In addition, it should be considered whether or not unfairness is undesirable in all situations. For example, in adult studies, intentionality of actions can be important in assessing unfair behaviours that deviate from the norm: People react less negatively to inequity when the unfair behaviour was not conducted intentionally [37]. Further, an fMRI study showed that activities in regions related to the understanding of intentionality, the temporoparietal junction and dorsolateral prefrontal cortex, mediated age-related changes in rejections of unfair offers in the UG [38]. Intentionality of immoral actions may influence evaluations about accepting unfair behaviours. Therefore, adults may use social information to interpret a prosocial character’s intention to make unfair distributions, and the unfair actions of the prosocial character may be considered as occurring in exceptional situations without immoral intention. In other words, participants may conclude that the prosocial character’s unfair behaviour is adventitious. Some developmental behavioural studies have suggested that children consider unfair distributions to be aversive starting from 7 to 8 years [39]. This may be related to the increase in the understanding of intentionality [40]. Although we did not control the intentionality of the distributions, the character with prosocial traits may have been accepted as making unfair distributions considering intentionality, so it may be acceptable for adults that the prosocial character, the Helper, acts unfairly. The development of fairness needs to be considered in light of the comprehension of intentionality.

Although we have provided some insights on fairness in adults, we evaluated only ERP without behavioural measurements, such as eye tracking. Many developmental studies have used eye gaze tracking to report a preference for prosocial information. Thus, to compare developmental differences, it is necessary to combine simultaneous measurements of eye gaze data with neuroscientific techniques. Also, our sample size is small, so it can be a limitation of this study.

The present study is the first use of ERP to examine the effects of social information on the sense of fairness in adults. We revealed that social information may affect judgement of actions that result in affective evaluation, and the sense of fairness may change in development. Our paradigm may apply to developmental studies, so further research comparing adults and infants is required. Longitudinal studies are also needed to examine the developmental stages of fairness and processing of prosocial information.

## Supporting information

S1 Data Individual data of ERPs in the test phase. (XLSX)
